# Prenatal diagnosis of distal 13q deletion syndrome in a fetus with esophageal atresia: a case report and review of the literature

**DOI:** 10.1186/s13256-022-03713-z

**Published:** 2022-12-27

**Authors:** Tomomi Kotani, Hiroyuki Tsuda, Yumiko Ito, Noriyuki Nakamura, Takafumi Ushida, Kenji Imai, Yukako Iitani, Kazuya Fuma, Yukako Muramatsu, Masahiro Hayakawa, Hiroaki Kajiyama

**Affiliations:** 1 Department of Obstetrics and Gynecology, Nagoya University Graduate School of Medicine, 65 Tsurumai-Cho, Showa-Ku, Nagoya, 466-8550 Japan; 2 Division of Perinatology, Center for Maternal-Neonatal Care, Nagoya University Hospital, Nagoya, Japan; 3 Department of Obstetrics and Gynecology, Japanese Red Cross Aichi Medical Center Nagoya Daiichi Hospital, Nagoya, Japan; 4 Department of Pediatrics, Nagoya University Graduate School of Medicine, Nagoya, Japan; 5 Division of Neonatology, Center for Maternal-Neonatal Care, Nagoya University Hospital, Nagoya, Japan

**Keywords:** Array comparative genomic hybridization, Esophageal atresia, Prenatal diagnosis

## Abstract

**Background:**

Chromosome 13q deletion syndrome shows variable clinical features related to the different potential breakpoints in chromosome 13q. The severely malformed phenotype is known to be associated with the deletion of a critical region in 13q32. However, esophageal atresia is a rare symptom and the relevant region is unknown. Thus, determining the association between accurate breakpoints and new clinical features is essential.

**Case presentation:**

A 28-year-old Japanese primigravid woman was referred for fetal growth restriction, absence of a gastric bubble, cerebellar hypoplasia, overlapping fingers, and polyhydramnios at 31 weeks gestation. At 38 + 0 weeks, she delivered a 1774 g female infant. The infant presented with isolated esophageal atresia (Gross type A), Dandy–Walker malformation, right microphthalmia, left coloboma, overlapping fingers, pleurocentrum in the thoracic vertebrae, reduced anogenital distance, and hearing loss. Her karyotype was diagnosed as 46,XX,del(13)(q32.1–qter) by amniocentesis, but array comparative genomic hybridization after birth revealed the deletion of 13q31.3–qter. At 48 days after birth, the infant underwent surgery for esophageal atresia and was later discharged from the hospital at 7 months of age.

**Conclusion:**

This case report and the literature reviews supports the previous findings on the pathological roles of haploinsufficiency of the *ZIC2/ZIC5* in Dandy–Walker malformation and the *EFBN2* haploinsufficiency in eye malformation and hearing loss. Furthermore, the possible involvement of *IRS2*, *COLA1*, and *COLA2* in eye malformation were identified. This is the first case of 13q deletion syndrome with esophageal atresia (Gross A), but it may be a symptom of VATER/VACTER association (vertebral defects, anorectal malformations, cardiac defects, tracheoesophageal fistula with or without esophageal atresia, renal malformations, and limb defects), as in the previous cases. These symptoms might also be associated with *EFBN2* haploinsufficiency, although further research is required.

## Background

Genotype–phenotype analyses have revealed several critical regions related to specific anomalies in 13q deletion syndrome [1]. Brown et al. categorized 13q deletions into three groups: group 1, proximal deletions not extending into q32; group 2, more distal deletions including at least part of q32; and group 3, most distal deletions involving q33–34 [2]. They also suggested that the severely malformed 13q phenotype results from the deletion of a critical region in 13q32 [3], which was later verified by others [4, 5]. However, genotype–phenotype correlations are still not completely understood. Since 13q deletion syndrome is exceedingly rare and presents as multiple phenotypic symptoms, the collection and analysis of findings regarding genotype–phenotype correlations are necessary to better understand this condition.

In the present report, the patient was an infant with a prenatal diagnosis of 13q deletion syndrome, defined as the deletion of 13q32.1–qter, consistent with the most severe form of group 2 deletions. The neonate exhibited several related symptoms, including fetal growth restriction (FGR), congenital anomalies of microphthalmia, Dandy–Walker malformation (DWM), overlapping finger, and esophageal atresia. Esophageal atresia is a rare symptom. The comparative genomic hybridization (CGH) array revealed a breakpoint of 13q31.3.

## Case presentation

**Fig. 1:**
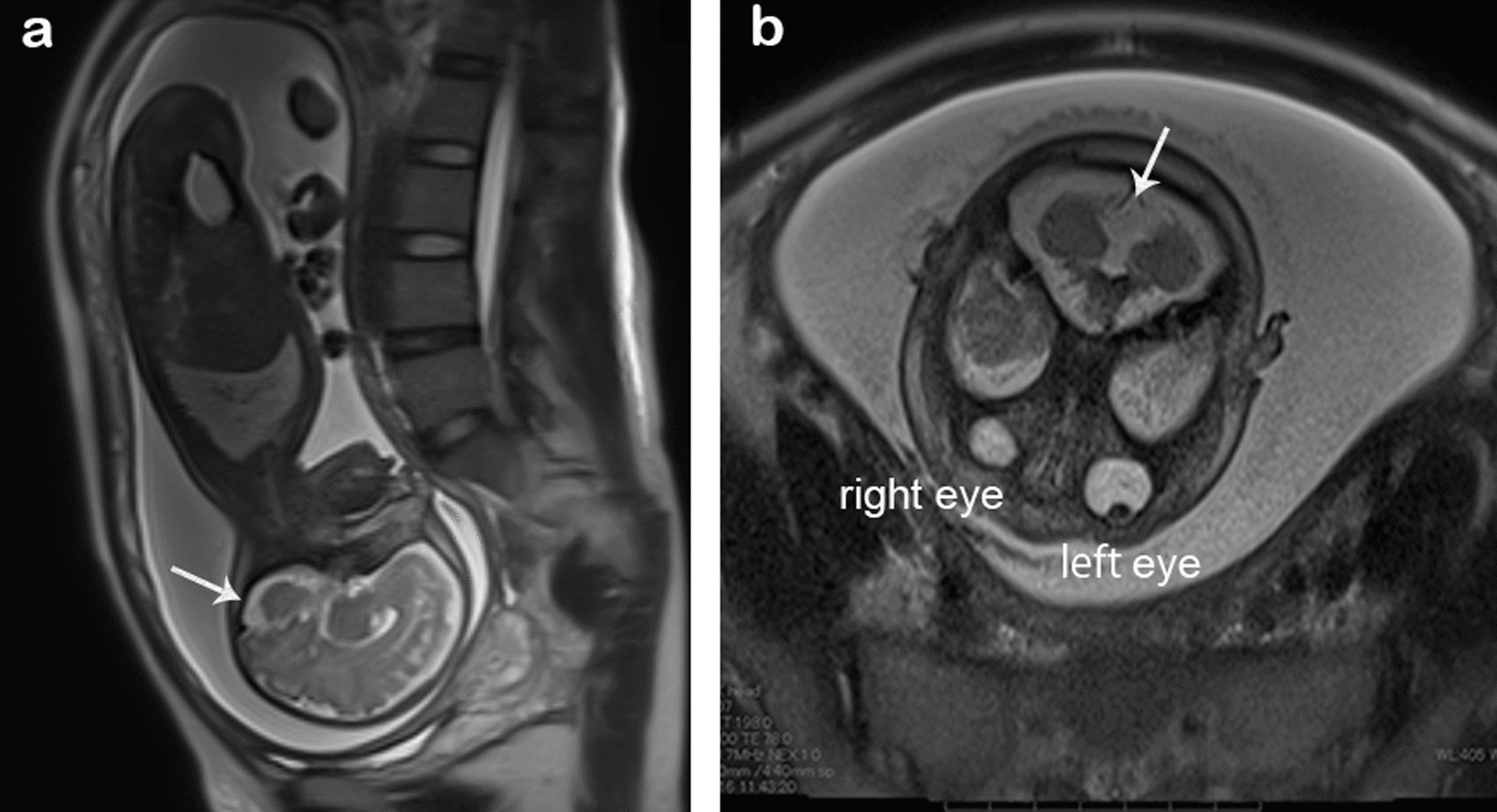
Fetal magnetic resonance imaging scans. Sagittal (**a**) and axial (**b**) views at 34 weeks of gestation. Arrows indicate the cerebellum

**Fig. 2:**
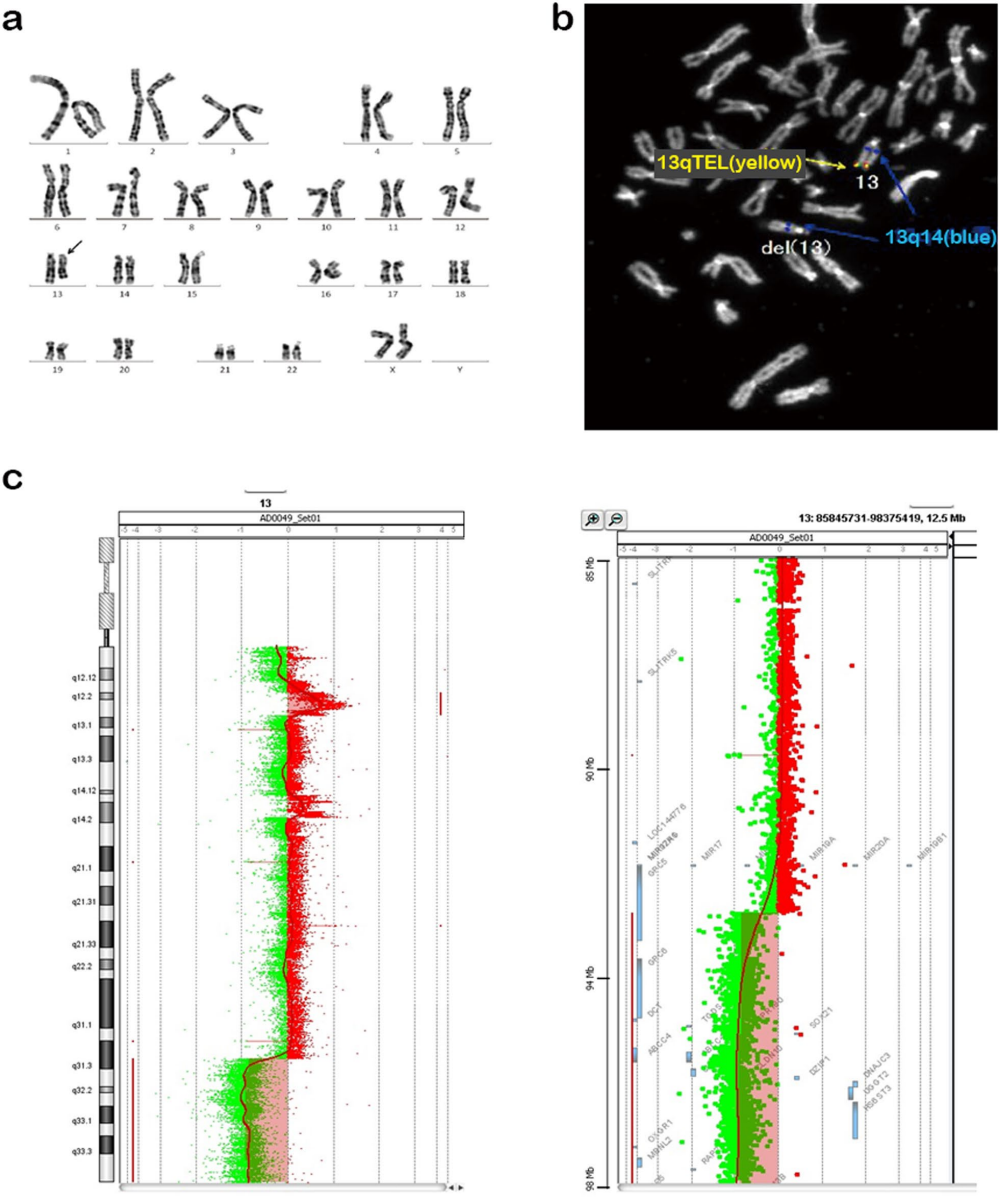
Chromosomal analyses. **a** Results of Giemsa banding by amniocentesis. The arrow indicates the abnormal chromosome 13, as del(13)(q32.1). **b** The result of fluorescent in situ hybridization by amniocentesis. The normal chromosome 13 had both 13q14 (blue) and 13qter (yellow), but no signal indicative of 13qter was detected on the chromosome del(13)(q32.1). **c** Array CGH analysis reveals the deleted region of 13q31.2–qter (92973314–115097664). The right panel shows an enlarged view of the gene deletion site in the left panel

A 28-year-old Japanese primigravid woman was referred at 31 weeks of gestation for abnormal ultrasound findings including FGR, absence of a gastric bubble, and polyhydramnios. She had no history of abortion or complications. The couple had no family history of genetic diseases. The fetus showed an estimated fetal body weight of −3.2 standard deviations at the first ultrasound survey, had a score of 26 on the amniotic fluid index, exhibited no gastric bubble, and had cerebellar hypoplasia (Fig. 1a, b, arrows) and overlapping fingers. Microphthalmia was also detected in the right eye on fetal magnetic resonance imaging (MRI) (Fig. 1b). Amniocentesis was performed, and Giemsa banding (Fig. 2a) and fluorescence in situ hybridization (Fig. 2b) revealed a 46,XX,del(13)(q32.1–qter) karyotype. At 38 + 0 weeks gestation, an infant girl weighing 1774 g was born and examined by pediatric doctors. She presented with isolated esophageal atresia (Gross type A), DWM, right microphthalmia, left coloboma, overlapping fingers, pleurocentrum in the thoracic vertebrae, reduced anogenital distance, and hearing loss. At 48 days after birth, the infant underwent radical surgery for esophageal atresia and was discharged from the hospital uneventfully at 7 months age. The CGH array (SurePrint G3 human CGH 1 × 1 M; Agilent Technologies, CA, USA) using villus sampling after birth revealed that the region stretching from 13q31.3 to the terminus was deleted (22.12 Mb; from 92,973,314 to 115,097,664 bp, GRCh37, Fig. 2c). The parents both had normal karyotypes, indicating that the infant’s 13q deletion was de novo. The couple later had three healthy children.

## Discussion and conclusion

**Fig. 3:**
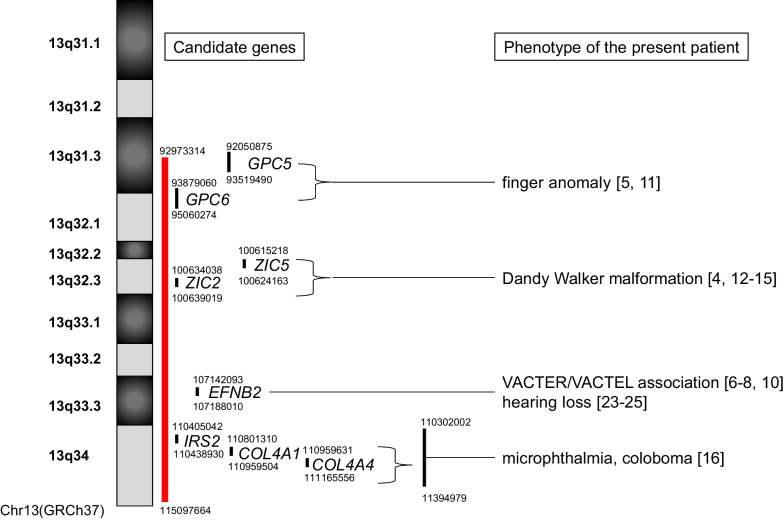
Candidate genes are related to the phenotype of the present patient. The red line shows the deleted region of the present patient. The numbers shown in the parentheses are consistent with the reference numbers

Esophageal atresia is a rare phenotype of 13q deletion syndrome. CGH array analysis determined that the breakdown point was 13q31.3. The patient also exhibited various phenotypes that matched with group 2 of 13q deletion syndrome, including overlapping finger, DWM, right microphthalmia, left coloboma, hearing loss, reduced anogenital distance, and pleurocentrum in the thoracic vertebrae (Fig. 3). She had no cardiac anomaly.

The relation between haploinsufficiency of *EFNB2* and vertebral defects (V), anorectal malformations (A), cardiac defects (C), tracheoesophageal fistula with or without esophageal atresia (TE), renal malformations (R), and limb defects (L) (VATER/VACTERL) associations in 13q deletion syndrome have recently been reported [6–8]. Esophageal atresia without tracheoesophageal fistula (Gross A), vertebra anomalies, and reduced anogenital distance in the present patient also indicate VATER/VACTERL association-like symptoms. We found no previous reports demonstrating esophageal atresia (Gross A) with 13q deletion syndrome, but esophageal atresia (Gross A) might be a symptom of VATER/VACTER [9]. Our review found three 13q deletion patients with esophageal atresia, including the present patient (Table 1) [8, 10]. All of these patients showed partial symptoms of VATER/VACTER. Therefore, the present patient and our literature review suggest that 13q deletion syndrome with esophageal atresia would show VATER/VACTERL association-like symptoms. Two of the three patients had 13.32q deletion, which results in the haploinsufficiency of *EFNB2*. However, the specific region associated with esophageal fistula/atresia remains undetermined. Therefore, further studies are needed to clarify the association between insufficiency of the 13q region and tracheoesophageal fistula/atresia. For this purpose, it would be helpful to determine the deleted region in detail using the CGH array.

**Table 1:** Comparison of clinical and chromosomal features in 13q deletion syndrome with esophageal atresia

	Jackson et al. [28]	Walsh et al. [25]	Present patient
Deleted region	13q12	13q31.1qter	13q31.3qter
Karyotype	46,XX,-13, + der(18)t(13;18)(q12;p11.2)	46,XY,del(13)(q31.1)	46,XX,del(13)(q32.1)
Deletion size	ND	ND	22.12 Mb
Parents karyotype	Normal	Normal	Normal
Gestational age at delivery (weeks)	36	39	38
Birth weight (g)	1500	1860	1774
Outcome	Stillborn	Death at 2 months due to to recurrent pulmonary and systemic infections	Discharged alive at 7 months
Esophageal atresia	Type C	Type C	Type A
Vertebral defects	Bilateral cervical ribs	Incomplete vertebral arch,multiple notched thoracic vertebrae	Pleurocentrum in the thoracic vertebrae
Anorectal malformations	None	Imperforate anus	Short anogenital distance
Cardiac defects	Small VSD	VSD	None
Renal malformations	None	None	None
Limb defects	None	Absent thumbs, hypoplastic radi	Overlapping fingers
Others	Poor skeletal ossification, persistent septum pellucidum anteriorly	Prenatal growth retardation, microcephaly, unusual facial features, penoscrotal transposition	DWM, right microphthalmia, left coloboma, hearing loss

*ND* not determined, *VSD* ventricular septal defect, *DWM* Dandy–Walker malformation

Finger anomalies are related to loss of the *GPC5* gene in 13q31.3 [5] and the *GPC6* gene in 13q31.3–q32.1 [11], consistent with the present patient. However, one report showed that limb abnormalities may not be related to any specific genomic region [12].

DWM is well known to be associated with haploinsufficiency of the *ZIC2* and *ZIC5* located in the 13q32.3 [4, 12–15], which is consistent with the findings of the present patient. In addition, the 13q33.1 may also be associated with DWM [14], which is also consistent with our patients.

Previous reports suggested that eye malformation might be associated with the *EFNB2* gene [4], and deletion of the 13q32 region [2]. Recently, the 13q33.3–q34 deletion (110,302,002–11,394,979, GPCh37) has been shown to be associated with microphthalmia or anophthalmia with/without coloboma in 15 patients [16], and 13 genes were encoded in the region: *IRS2*[110405042–110438930], *COL4A1*[110801310–110959504], *COL4A2*[110959631–111165556], *CARS2*[111293757–111358862], *ING1*[111364970–111375686], *SOX1*[112721463–112726020], *ATP11A*[113344352–113541482], *MCF2L*[113623528–113754056], *F7*[113760102–113774999], *F10*[113777113–113803843], *PROZ*[113812962–113826700], *PCID2*[113831850–113862983] and *CUL4A*[113862507–113921422] (GpCh37) [7]. In our review of these genes, *IRS2* was reported to be involved in retina function [17, 18]. *COLA1* and/or *COLA2* mutations are involved in ocular defects [19–22]. The present patient and our literature review suggest that *IRS2*, *COLA1*, and *COLA2* in the 13q33.3–q34 would also have a pathological role in eve malformation, including microphthalmia or anophthalmia with/without coloboma.

It has been reported that *EFNB2* haploinsufficiency [23–25] or 13q32 deletion [15], is involved in hearing loss, although deafness has been reported in the patients with deletions of the 13q13.1–q14.3 and 13q12.3–q21.1 [26]. Furthermore, *EFNB2* is involved in the morphogenesis of the endolymphatic sac and duct epithelia in the mouse inner ear, which requires normal hearing [27]. Our data also supports the role of the *EFNB2* haploinsufficiency in hearing loss.

The present patient showed no cardiac defect without the distal 13q34 region, which has been reported to be associated with cardiac defect [7, 28].

This study presented a case that indicated an association between esophageal atresia, a rare phenotype, with deletion of the 13q31.3–qter region. The present case and the literature review suggest that it is part of VATER/VACTERL association-like symptoms and suggests the association between haploinsufficiency of *EFNB2* and VATER/VACTERL association-like symptoms. These findings support the previous findings on pathological roles of haploinsufficiency of *ZIC2/ZIC5* on DWM, and *EFBN2* haploinsufficiency on eye malformation and hearing loss. Furthermore, we identify the possible involvement of *IRS2*, *COLA1*, and *COLA2* in eye malformation. We hope that these findings will help identify the causal genes of various phenotypes of 13q deletion and provide more precise information during prenatal counseling, although further accumulation of such reports is required.

## Data Availability

Not applicable.
